# Levetiracetam for the Prophylaxis of Migraine in Adults

**DOI:** 10.7759/cureus.16779

**Published:** 2021-07-31

**Authors:** Gauhar M Azeem, Filzah Faheem, Nimrah Farooq, Danish Sohail, Abdul Rehman, Muhammad Usama Javed

**Affiliations:** 1 Neurology, Services Hospital, Lahore, PAK; 2 Psychiatry, Services Hospital, Lahore, PAK; 3 Internal Medicine, Jinnah Hospital, Lahore, PAK; 4 Internal Medicine, Faisalabad Medical University, Faisalabad, PAK; 5 Medicine, Services Hospital, Lahore, PAK; 6 Medicine, Faisalabad Medical University, Faisalabad, PAK

**Keywords:** migraine, levetiracetam, prophylaxis, adult, review

## Abstract

Migraine is the second most common primary headache disorder after tension-type headache and is the leading cause of disability worldwide. Cortical spreading depression involves neuronal excitation and inhibition and is involved in pathophysiology of migraine. Many anti-epileptic drugs act by inhibiting Cortical Spreading Depression and block desensitization. Anti-convulsants are commonly used in Migraine prophylaxis and the ones being more effective than placebo include Sodium Valproate and Topiramate. Levetiracetam has unique mechanism as it targets hyper-excitable neurons by binding to synaptic vesicle protein. This results in inhibition of neurotransmitter release thus decreases hyper-excitability. Levetiracetam has minimal side effect profile (dizziness, somnolence and mood changes) and it can be well tolerated by patients. In this review, a total of seven studies were included (four open-label trials and three randomized-control trials) which evaluated the role of Levetiracetam in the prophylaxis of migraine in adult patients. On review of this evidence, Levetiracetam appears to be effective in treating migraine with and without aura and is considered safe because of its limited side effects. There was a significant reduction in the frequency, severity, and duration of migraine with a high responder rate. Levetiracetam was well tolerated with minimal side effects and no reported interactions. However, larger randomized controlled trials are needed and these studies should be done on special population to see the outcomes. In addition, studies for extended-release formulations should also be done.

## Introduction and background

Migraine is the second most common primary headache disorder after tension-type headache and is the leading cause of disability worldwide [1]. The most common types of migraine include migraine without aura (66%) and Migraine with aura (33%) [2]. It is a highly prevalent headache disorder affecting about 37 million people in the United States and one billion people worldwide [3]. Incidence of migraine was found to be 8.1/1000 people in a follow-up epidemiology study [4]. In adults Migraine is three times more prevalent in females than males, being more prevalent in 20-40 years of age [5,6]. A cross-sectional prevalence survey conducted in Pakistan revealed the overall prevalence of migraine to be 22.9% most common in 40-49 age group (20.6% in males and 38.3% in females) [7].

Sensory threshold changes in hypothalamus during premonitory phase and thalamo-cortical activity during migraine attack results in aberrant symptoms like allodynia and photophobia. Calcitonin gene-related peptide (CGRP) and pituitary adenylate cyclase-activity polypeptide (PACAP) serve as migraine mediators and therapeutic targets [8]. Effective response to non-vasogenic medications has put question mark on vasogenic theory of migraine as many patients failed to respond to ergotamine-derived triptans. In addition, positron emission tomography scan during episode of migraine highlighted blood flow inconsistent with neurovascular distribution [9]. Cortical spreading depression involves neuronal excitation and inhibition and is involved in pathophysiology of migraine. Many anti-epileptic drugs act by inhibiting Cortical Spreading Depression and block desensitization [10].

Prophylactic drugs are used to decrease the number of migraine attacks and to prevent them from happening. Prophylactic drugs being used for migraine include beta-blockers, alpha-blockers, anti-convulsant, serotonin agonists, selective serotonin reuptake inhibitors (SSRIs), TCAs and CGRP antagonists among others [11]. Both migraine and epilepsy go hand in hand, it has been observed that children with migraine also had epilepsy, similarly, people diagnosed with epilepsy have higher rates of migraine when compared to general population data [12] as both diseases involve excitation of neocortical cells. CSD, glutamate hyperexcitability and trigeminovascular pain pathological mechanisms connect both migraine and epilepsy. A strong link between post-traumatic, partial, cryptogenic epilepsy and migraine has been observed. Seizures-related headaches such as migralepsy, ictal epileptic headache and hemicrania epileptica further validate the association between migraine and epilepsy [13].

Anti-convulsants are commonly used in migraine prophylaxis and the ones being more effective than placebo include Sodium Valproate and Topiramate [11]. Topiramate and Sodium Valproate are approved drugs for migraine but their teratogenic effect limits their use in pregnant patients, have multiple drug interactions and can cause dose-dependent side effects. In comparison to other anti-epileptic drugs, Levetiracetam has unique mechanism as it targets hyper-excitable neurons by binding to synaptic vesicle protein. This results in inhibition of neurotransmitter release thus decrease hyper-excitability. It can also inhibit N-type calcium-channels. Levetiracetam has a minimal side effect profile (dizziness, somnolence and mood changes) and it can be well tolerated by patients [10]. Its role in the prophylaxis of migraine in adults has been investigated for two decades now and this is a review of the currently available evidence.

## Review

Electronic literature search was done on PubMed, Elsevier, Google Scholar and Cochrane using keywords like Levetiracetam and Migraine to include maximum possible results. PubMed showed 78 results concerning our question, all the results were read in detail and out of these results we found three randomized controlled trials and three open-label studies contained direct evidence pertaining to our question and they were included in the review. After a study of three meta-analysis and nine review articles, we found one more open-label study relevant to our interest. Two results relevant to our question were found on Cochrane, one result was the same as found on PubMed.

Results

Only seven studies were included in this review after thoroughly reviewing researches and removing duplication.

A review study done by Li et al. in 2018 included 25 patients, out of which 11 either did not follow up or dropout, so, 14 patients (12 females and two males) with migraine between 15 and 60 years of age (mean age 40.19 years) who satisfied the International Classification of Headache Disorders, 3rd edition (ICHD-III) criteria completed the study. They had one or more migraine attacks/month as declared by the physician and duration of migraine ranged from three to 30 years. Thirteen subjects had migraine with aura while one had migraine without aura. LEV was started at 250 mg/day in the first week and increased to a total dose of 500 mg/day (250 mg, B.D) after one week. Magnetic Resonance Spectroscopy was repeated and headache frequency and duration were noted after 12 weeks of therapy and patients did not take any medication except ibuprofen. Patients on Levetiracetam treatment showed a significant decrease in frequency and duration of migraine. Out of 14 patients who had re-examination for MRS after receiving LEV therapy for 12 weeks, 11 of these patients showed GABA + in posterior cingulate cortex to be of good quality, eight had GABA + in anterior cingulate cortex/median prefrontal cortex. In posterior cingulate cortex regions, GABA+ levels decrease indicated brain chemistry changes to be related to migraine and effectiveness of LEV [14].

A small open-label trial conducted by Pizza in 2011 treated 13 elderly patients (eight females and five males, aged 60-72 years mean age 64.7 years ±3.4) who suffered from migraine without aura with duration of disease two to 45 years with mean value of 21.3 years ±13.4). They used ICDH’04 migraine criteria. None of the patients took migraine preventive therapy and they had migraine for at least one year but all patients took chronic disease medications. After recruitment Levetiracetam 500 mg/day was administered for one week and 1000 mg per day for six months. Baseline migraine frequency noticed was 12.2/month (SD 5.9) and it decreased to 8.3 (SD 4.9), 4.1 (SD2.6), 1.3 (SD1.4) at one, three and six months, respectively [P = 0.079; P < 0.0001]. Seven (54%) patients experienced somnolence, decreased concentration and stomachache. Drug was well tolerated by patients and no one withdrew from the study. Twelve of 13 patients who were receiving treatment for other comorbidities did not experience any drug interaction [15].

A six-month open-label study conducted by Brighina in 2006 included 16 patients (11 females and five males) experiencing migraine with aura. Patients included in study had four or more episodes of migraine per month that should be experienced for at least three months aged 16-59 years with no previous complain of epilepsy or mood disorder. All the patients in study received 1000 mg of Levetiracetam (500 mg twice a day) for six months. Initially, dosage of 250 mg/d and then dosage increased 250mg per week until the 1000 mg/d was achieved. During the study period, they used paired t-test and a comparison between severity and duration was made. Out of 16 patients, seven (44%) became headache-free by three months while nine (66%) patients experienced monthly decrease. Seven patients had no aura symptoms after receiving treatment for three months while remaining had significant decrease in the mean duration of aura (p < 0.001) during the third month of treatment (11.4±6.2min) and sixth month of treatment (16±8.6 min) while in run-in period (41.3±14.4 min). Frequency of attacks reduced from (6.20±1.6) to (0.81±0.8) till the end of six months of treatment. The adverse effects reported by patients only during the titration phase included somnolence in two patients, dizziness in one patient, and nervousness in three patients [16].

Open-label study conducted by Gallai et al. in 2003 included 20 patients (seven men, 13 women; all were >18 years with mean age, 43.5 years±13.2 years) having migraine without aura for at least one year, were diagnosed according to 1988 criteria of IHS. Inclusion criteria were four migraine attacks/month but should not be greater than 15 days/month (to exclude Chronic Migraine), symptomatic drugs intake for attacks should not exceed or fulfil the current IHS criteria defining drug abuse. Levetiracetam was initiated 500 mg HS, increased by 500 mg after every five days, until it reached 2000 mg/day then no further changes in were made. Patients were re-assessed on monthly basis to assess the headache diary and related symptoms. Only one patient dropped out. At the start of study, patients experienced moderate or severe headache (mean of 10.7 days/month). After taking treatment for three months, 11 (57.9%) experienced reduction of at least 50% and frequency also significantly reduced (p<0.001) in these patients. Even after discontinuation of levetiracetam carry over effect remained for three months in those 11 patients. Nine patients (81.8%) had short time period carry-over effect (one month post drug suspension) and seven (63.6%) experienced continuous relief (three months after suspension of drug). The mild adverse effects reported were somnolence by 68.4%, body weakness by 47.3%, and unstable posture by 31.5%; patients tolerated them well [17]. Of note, the dose used in this study was 2000 mg/day and higher than those in other studies.

A randomized placebo-controlled double-blinded study performed by Sadeghian in 2015 included 85 migraine patients (aged 20-52 years), eight with aura and 77 without aura according to ICHD-II criteria. Twenty-seven patients in Levetiracetam group were started on an initial 250 mg/day of levetiracetam, increased to total dose of 500 mg/day, 32 subjects in Valproate group were prescribed 500 mg/day of Sodium Valproate and 26 subjects were started on placebo. The study was designed in a double-blinded fashion. The patients were assessed at one, three and six months. Both Levetiracetam (63%) and Valproate (65%) had significantly reduced (>50%) attacks after duration of six months as compared to placebo (P = 0.019 and 0.012, respectively), while no significant difference was noted between Levetiracetam and Valproate (P = 0.78). Twenty-three patients (85.2%) on Levetiracetam and 22 (68.8%) in Valproate group experienced no side effects. The most common side effects in Levetiracetam patients were somnolence, dizziness, irritable mood, hostility, and hyperactivity [18].

A prospective, randomized, placebo-controlled trial carried out by Verma in 2013 included 52 patients (38 females & 14 males, aged 17-46 years, mean age 30.01 years) having migraine with or without aura. Patients had four or more attack/month for duration of minimum three months and they failed or stopped previous medications due to side effects. The inclusion criteria were re-assessed at the end of run-in period. Subjects were randomly assigned to Levetiracetam or placebo group (ratio was 1/1). Levetiracetam was started at a dose of 250 mg/d, was increased weekly until the dosage of 1000 mg/d was achieved. Patients were followed-up on monthly basis to assess frequency, severity, clinical disability, acute medication taken for severe migraine, and adverse events as recorded in diaries by the patients. 13 patients left the trial, 25 patients were included in the Levetiracetam group and 27 patients in the placebo group. The migraine duration was one to 20 years. Mean frequency significantly reduced from 5.17 (SD, 1.19) at to 2.21 (1.47) in the last four weeks in patients who were in Levetiracetam group ( P <0.0001), as compared to placebo (P = 0.088). 64% patients treated with Levetiracetam showed ≥50% reduction in migraine frequency while 22% of patients in the placebo group. Decrease in migraine disability (on MIIDAS Score) in both the Levetiracetam (3.33 [0.81] to 1.66 [0.76], P < 0.0001) and placebo group (from 3.19 [0.94] to 2.38 [0.94], P < 0.0025) was noticed being more significant in Levetiracetam group. Symptomatic drugs intake reduced from 5.85 (1.55) to 1.87 (1.39) tablets per month (P < 0.0001). No response difference between migraine with or without aura was observed [19].

Double-blind, controlled study performed by de Tommaso in 2008 included 45 patients (35 females and 10 males) having migraine without aura. All patients were eligible for migraine prophylaxis and they aged between 18 and 49 years with mean age 37.86 ± 12.35. They were randomly given Topiramate (50 mg) twice a day, Levetiracetam (500 mg) twice a day or placebo. While one patient was given placebo, two patients randomly received either Topiramate or Levetiracetam. Twenty-four healthy, non-migraine subjects [eight males and 17 females, aged 18-48 (mean age 35.2 ± 5.56)] were also included for EEG evaluation. They compared headache frequency in the two months before and after the treatment. Information was taken from a diary in which patients were guided to note headache occurrence, frequency and duration. Headache frequency and duration were the same in the three randomized groups. Six patients (four in the placebo group and two in the topiramate group) dropped out as they were non-compliant and one patient (topiramate group) left as a result of side effects such as drowsiness and sedation. Eight patients in Levetiracetam group experienced migraine frequency <50% of the basal frequency, five had 55-65%, and two had 85-95%. In comparison to placebo, both Levetiracetam and Topiramate reduced the frequency of headache. Difference between Levetiracetam and Topiramate was not significant. Five patients in Levetiracetam group experienced sedation and dizziness in the initial days of therapy. However, all side effects were well tolerated and did not result in therapy cessation (Table 1) [20]. 

**Table 1:** Summary of results.

Study	Participants, and dose	Study type	Migraine type, age of participants	Outcome	Key results	Study weakness
Li et al. (2018) [14]	14, levetiracetam 500 mg/day	Open label	Migraine with aura and migraine without aura, 15-60 years	Frequency decreased, responder rate not mentioned	Significant	Small sample size, clinical efficacy not primary goal, high risk of bias
Pizza et al. (2011) [15]	13, levetiracetam 1000mg/day	Open label	Migraine without aura, On multiple other medication, 60-72 years	Frequency decreased, responder rate not mentioned	Significant	Small sample size, high risk of bias
Brighina et al. (2006) [16]	16, levetiracetam 1000 mg/day	Open label	Migraine with aura, 16-59 years	Frequency decreased, 44% completely headache free	Significant	Small sample size, high risk of bias
Gallai et al. (2003) [17]	19, levetiracetam 2000 mg/day (all previous non responders)	Open label	Migraine without aura, mean age 43.5 years	Frequency decreased, 57.9% responders	Significant	Small sample size, high risk of bias
Sadeghain et al. (2015) [18]	27 levetiracetam 500 mg/day, 32 Valproate 500 mg/day, 26 placebo	RCT	Migraine with aura and migraine without aura, 20-52 years	Frequency decreased, 63% responders	Significant against placebo, result not significant against Valproate	Small sample size
Verma et al. (2013) [19]	25 levetiracetam 1000mg/day, 27 placebo (failed previous prophylaxis)	RCT	Migraine with aura and migraine without aura, 17-46 years	Frequency decreased, 64% responders	Significant	Small sample size, high drop out rate
de Tommaso (2008) [20]	15 levetiracetam 1000 mg/day, 12 topiramate 100mg/day, 11 placebo	RCT	Migraine without aura, 18-49 years	Frequency decreased, 53.3% responders	Significant, result not significant against Topiramate	Small sample size, clinical efficacy not primary goal of study, follow up only two months

Discussion

Levetiracetam appears to be effective in treating migraine with and without aura and is considered safe because of its limited side effects. In this review, a total of seven studies were included (four open-label trials and three randomized-control trials). Among three randomized trials, two included patients with both migraine with aura and without aura [18,19] and one included patient without aura [20]. While among four open label trials, two included patients with migraine without aura [15,17], third one included patient with aura [16] and the fourth one included patient with aura and without aura [14]. Five out of seven studies mentioned use of migraine criteria (ICHD-II, ICHD-III, ICHD IV & HIS) [14-18] while other studies used migraine attack frequency and associated symptoms to be diagnosed by physicians [19,20]. Age of patients included in the studies ranged from 15 to 72 years.

 All studies (four open-label trials and three retrospective trials) mentioned significant decrease in migraine frequency. Five studies that mentioned the responder rate (>50% reduction in frequency of migraine) to Levetiracetam ranged from 44% to 64% [16-20]. In the remaining two studies done to assess Levetiracetam effectiveness; responder rate was not mentioned [14,15]. Patients who used Levetiracetam for migraine reported maximum effect in reducing migraine frequency at three months of treatment while only one study showed Levetiracetam to be most effective even after one month of treatment [17]. Patients continued to experience decrease in migraine frequency over the next months of treatment who were not able to have achieved headache-free period after three months of treatment.

Six researches out of seven discussed decrease in migraine attack frequency that ranged from 0.81-2.9/month on completion of study duration from baseline representing significant reduction in migraine frequency [15-20]. All these data support Levetiracetam to be effective to decrease migraine frequency > 50%. Total number of participants included in studies was 245. Only six studies provided data about female (117) and male (43) participants [14-17,19,20] while no gender difference in results and efficacy of Levetiracetam was observed. Only five studies reported drop out of patients during study time period but none dropped due to Levetiracetam medication adverse effects [17-20]. Studies that mentioned the duration of migraine, it ranged from one month to 45 years [14-15,18-19]. Starting dosage of Levetiracetam ranged from 250-500mg and it was titrated on a weekly basis until it reached maximum dosage of 500-2000 mg orally (O.D or B.D) and beneficial effects were observed on each dosage.

Only two studies reported intake of previous migraine prophylactic medications by patients [17,19] and two reported intakes of symptomatic drugs by patients [14,16] while one reported intake of chronic disease medications by subjects [15]. Levetiracetam was effective to treat migraine in patients who had failed to respond to other FDA [17] approved prophylactic medications and also no drug-drug interactions were observed in patients already taking medications for other chronic medical disorders. Two studies used comparator (Valproate, Topiramate) to see efficacy of Levetiracetam against them, they reported no significant difference between Levetiracetam against Valproate 500 mg OD and Topiramate 50mg BD [18,20].

A double-blinded placebo-controlled trial to study efficacy of Levetiracetam in treating chronic daily headache done in 2010 failed to show effectiveness of Levetiracetam 3000mg in treating headache. Important point to be considered here is that Levetiracetam is effective in treating migraine but failed to decrease chronic headache maybe signifying mechanism of action of the drug related to migraine pathophysiology but not chronic headache. This needs more data study and more large population studies to understand the mechanism and significance of Levetiracetam in treating migraine [21].

 In all studies included in this review, Levetiracetam is found to be significantly effective in decreasing frequency, duration, the severity of migraine attacks and is safe with limited side effect profile. Most effects seen by patients include Somnolence, dizziness, irritability, nervousness, mood change, postural instability [14-20]. These side effects were not severe enough to cause drop out of patients and they mostly occurred titration phase of treatment and no drug-drug interaction limited its use in chronic disease patients. All patients completed treatment period without experiencing serious side effects that could have resulted in stoppage of drug intake.

One common issue in all studies was the relatively small sample size of the studies which in turn increases the risk of bias. However, the results were generally similar when considering efficacy and tolerability and were statistically significant in all studies.

## Conclusions

The question about the efficacy of Levetiracetam in prophylaxis of migraine has been in discussion for about two decades. Researches have been done on this, but they are few and far between. Levetiracetam is found to be effective in migraine headaches (migraine aura and without aura) when compared with placebo, frequency duration and severity of migraine episodes decreased significantly, there was less use of acute treatments and high responder rate was observed. In studies, dosage ranged from 500 to 2000 mg per day and maximum effect was observed after intake of Levetiracetam for three months. Improvement was seen in treatment-naive patients and those who had failed previous treatments. It was well tolerated by patients, only mild side effects such as somnolence, dizziness and irritability were commonly seen in patients. Studies done to see the efficacy of Levetiracetam were small and there was high risk of bias.

Thus, Levetiracetam for migraine prophylaxis is effective as per current evidence. It can emerge as great, effective and safe option for many. However, larger randomized controlled trials are needed and these studies should be done on special population to see the outcomes. In addition, studies for extended-release formulations should also be done.
